# Bloody Vesicle of the Vagina

**Published:** 1847-04

**Authors:** John A. Cotten

**Affiliations:** Greenwood, Mississippi


### FACTS FROM CORRESPONDENTS.

---

### BLOODY VESICLE OF THE VAGINA.

By John A. Cotten, M.D., of Greenwood, Mississippi.

July 7th, 1837, Dr. Stokes and myself were called to see a servant girl of Mr. E. H. Stone, then of Madison county, Miss. The patient was aged about 23 years, decidedly above ordinary size, well proportioned, and of sound constitution. She was between seven and eight months advanced in pregnancy, and complained of an incessant itching and burning sensation at the vulva, accompanied by general constitutional disturbance. Upon examination, we found situated within the vagina, about one inch from the sphincter, an exceedingly vascular tumor, very elastic, of an oval form, and about one inch in diameter. The woman informed us that the growth had been very gradual, and the uneasiness proportioned to its size, the pain increasing with the growth of the tumor. To be more specific, the tumor had been about a month acquiring the size to which it had attained when we saw it. In appearance and consistency it resembled more than anything else a bloody vesicle filled to its greatest capacity. Thinking as we did that the gravid uterus afforded the best rationale of the complaint, we took from the arm of the patient about twenty ounces of blood, ordered a saline cathartic, cold astringent washes to the parts, low diet and the recumbent posture. This plan of treatment was persisted in for about ten days, without checking in the least the progress of the disease.

Having failed in this attempt to disperse the tumor, and no pulsation being perceptible, it was proposed to evacuate the tumor by puncture, and accordingly Dr. Stokes made a small opening with a thumb lancet. A stream of dark venous blood, of fully the size and force of that in venesection from the arm, followed the operation. We suffered the blood to flow to the extent of about ten ounces, without the slightest diminution in the size of the tumor, the supply being fully equal to the loss. We began now to suspect that the tumor was aneurismal in its nature. All efforts to arrest the flow of blood by compression proved abortive. I finally proposed strangulation with the ligature, and, Dr. Stokes concurring in the suggestion, I immediately passed a curved needle, armed with a strong thread, below the base of the tumor, and divided the ligature so as to embrace the posterior half with one portion of the thread and the anterior with the other. The ligature was tightly drawn around the respective portions of the tumor, and the hemorrhage immediately ceased.

On the fourth day the strangulated portion sloughed, and the patient appeared to be doing well for a few days; but in a little while the same itching, burning sensation which characterized the pain of the first tumor began to manifest itself still higher up the vaginal canal. Careful examination brought to view another tumor of the same appearance and about half the size of the first. After much difficulty, we succeeded in embracing with a double ligature the base of this tumor also, as we had practised in the first. In due time it sloughed, and all unpleasant symptoms subsided. At the full term, our patient gave birth to a well grown and healthy child.

It is now more than nine years since the removal of the tumors, and though the woman has given birth to three or four children since, there has been no return of the disease.

January 25, 1847.

